# Health fee exemptions: controversies and misunderstandings around a research programme. Researchers and the public debate

**DOI:** 10.1186/1472-6963-15-S3-S4

**Published:** 2015-11-06

**Authors:** Jean-Pierre Olivier de Sardan

**Affiliations:** 1 Laboratoire d'Etudes et de Recherche sur les Dynamiques Sociales et le Développement Local (LASDEL), BP 12901, Niamey, Niger; 2 École des Hautes Etudes en Sciences Sociales (EHESS), Paris, France

## Abstract

Our research programme on fee exemption policies in Burkina Faso, Mali and Niger involved sensitive topics with strong ideological and political connotations for the decision-makers, for health-workers, and for users. Thus we were confronted with reluctance, criticism, pressures and accusations. Our frank description of the shortcomings of these policies, based on rigorous research, and never polemical or accusatory, surprises political leaders and health managers, who are accustomed to official data, censored evaluations and discourse of justification.

This reflexive paper aims to react to some misunderstandings that arose regularly: "By focusing on the problems, you will discourage the aid donors". "By focusing on the problems, you are playing into the hands of the opponents of fee exemption". "You should focus on what works and not on what doesn't work". "The comments and behaviour you report are not representative". "What you say is not new, we already knew about it".

Double discourse prevails in aid-dependent countries. The official discourse is mostly sterilized and far removed from reality. It protects the routine of the local bureaucracies. But the private 'speak' is quite different, and everyone knows the everyday ruses, tricks and arrangements within the health system. Anthropologists collect the private speak and transmit it to the public sphere through their analyses in order to provide a serious account of a reality, and creating the conditions for an expert debate and a public debate. The national conference on fee exemption held in Niamey in 2012 was a success in this perspective: healthcare personnel spoke for the first time in a public setting about the numerous problems associated with the fee exemption policy, and they largely confirmed and even supplemented the results of our research.

It is difficult to see how the healthcare system can be improved and better quality of service provided without starting from a rigorous diagnosis of these usually concealed realities. Such diagnosis gives arguments to reformers within the health system to make change happen.

## Introduction

Our research programme on fee exemption policies in Burkina Faso, Mali and Niger has revealed many shortcomings, bottlenecks, and unexpected effects due to the implementation process of these policies [1]. Studying any policy implementation always discloses some implementation gaps. In the context of post-colonial and aid dependent States, such gaps are wider and more numerous [2]. But, paradoxically, they are less put on the table and scarcely discussed in the public sphere.

Such a context may explain why, throughout the duration of our research, we were confronted with a variety of reactions including reluctance, criticism, pressures and accusations. Let us consider two opposite examples. In Niger, we were often considered as "anti-exemption", such allegations originating from contexts related to institutions concerned by the fee exemption measures (Ministry of Health of Niger, AFD - French development agency -, WHO, and some NGOs) and reflect the political challenges associated with the measure. For example, following the distribution among health policy decision-makers in Niger of a policy brief that we have published on the serious problems encountered by the implementation of the fee-exemption policy at the level of the CSIs (*Centres de santé intégrés*, integrated health centres), we received a letter of strong denial from the Ministry of Health (April 2010), maintaining that there were no problems: the decision to implement fee exemption had been taken "in complete sovereignty", no partners had been "presented with a *fait accompli*", the financing was normal and programmed, the population was "informed and made aware", and there had been "an effective involvement of the healthcare personnel and management committees". All these statements are in total contradiction with our data. In Mali, on the other hand, healthcare personnel believed that our team had been commissioned by the state or the aid organizations to audit the fee exemption system and flush out illicit practices.

Most of the arguments opposed to us can be summarised in few sentences: *"*By focusing on the problems, you will discourage the donors"; "By focusing on the problems, you are playing into the hands of the opponents of fee exemption"; "You should focus on what works and not on what doesn't work"; "The comments and behaviour you report are not representative. These are exceptions which you blow up out of all proportion"; "What you say is not new, we already knew about it". We will discuss and contest these arguments one by one in this paper. Indeed, our research programme addressed sensitive topics with strong ideological and political connotations (concerning for instance the inconsistencies of public policies, aid-dependency, opposition between 'pro gratuity' and 'pro cost-recovery' factions, informal payments in health facilities...), involving many actors: decision-makers at national and international levels, health staff, NGO's militants in the health sector, and sometimes, also researchers themselves. Of course, we ultimately intended to assist those responsible for public policies relating to fee exemption in the three countries to improve these policies. To this end, a rigorous diagnosis of the associated problems, irrespective of their nature, was required and this is what we did. However, when expressed by independent research, this frank language (albeit not polemical or accusatory), based on serious research (irrespective of the method used, see Ridde & Olivier de Sardan, in this issue), generally surprises political leaders and managers who are accustomed to consulting data provided by official services or commissioning studies and evaluations by consultants, many of whom strictly limit themselves, not unexpectedly, to the perspective and framework defined by their commissioning parties [3]. In both cases the resulting analyses are not truly independent and are frequently subject to both censorship and self-censorship. Moreover, the language of political leaders and the representatives of development agencies generally tends to justify the actions carried out once the decisions are made and to underestimate the difficulties encountered: it would not be incorrect to refer to a 'culture of camouflage' here. Finally, the strong degree of personalization of the fee exemption policy around the President in power gave rise to various fearful and defensive reactions on the part of actors who felt that they were being called into question by the description of problems systematically erased from the official discourse.

Irrespective of the people who expressed their reservations or disagreement, the arguments and rumours that were levelled against us researchers deserve attention because they are indicative of certain tensions within the national and international institutional apparatuses which define and implement public policies, and of the misunderstandings that regularly arise between researchers and decision-makers.

Hence, we shall take these misunderstandings seriously here and attempt to respond to them based on the reactions to our research on the fee exemption policies and also, more generally, on the experience of LASDEL in Niger since 2000 [3].

## "By focusing on the problems, you will discourage the donors"

This is an argument that has often been made to us, not only during this research project but also in the course of other LASDEL programmes. It is indicative of the dependency of the national contexts on development aid and their concern with "pleasing the donors" and presenting as "good pupils" of the international institutions [4]. This kind of strategy can be observed from the top to the bottom of the chain for capturing the 'development rent': a very often false 'community-based' harmony is 'staged' in the villages with a view to attracting 'a project'; at the top level of the State, reports are written which extol the measures implemented and ignore the difficulties encountered and, more generally, the actual impacts of the measures.

This strategy of concealing problems makes the opposite approach of documenting them, as we do, appear 'scandalous'. During a presentation of the results of our research programme in Bamako, we were accused of "tarnishing the image of Mali" for simply having reported the following statement made by a healthcare worker (and one that is no doubt regularly uttered by many Malians): "Nothing is taken seriously here in Mali and this explains the negligence, lack of will and absence of reliability in supplying the ASACOs (community health associations) with sufficient and complete ACTs (artemisinin-based combination therapy, against malaria)" [5]. Simply speaking about the reality that prevails in this sector is equated with striking a low blow that will cut off external rent. Some people even believe it to involve a concealed audit on the part of the aid agencies or a police enquiry. Hence LASDEL was accused of having dissuaded the AFD from continuing to provide support to Niger in this sector due to its analysis of the negative impacts of the fee exemption policy.

This is to misjudge the nature of the decision-making process within the aid agencies. The belief that a report by LASDEL would be read by the senior managers of the development institutions and would directly affect their decisions is indicative of a profound ignorance of this world: the granting or extending of budget support is the responsibility of the government and not of the technical staff, and such decisions are based primarily on political criteria and not technical ones. Moreover, the belief that the technical and financial partners are naive to the point of misjudging the problems is itself naive. 'Camouflaging' is a strategy that may eventually backfire on those who practise it: 'camouflaged' problems eventually come to light in the public sphere and those responsible for concealing them are discredited. Finally, to the extent that the negative impacts revealed by us were mainly caused by the lack of financing of the fee-exemption policy, the conclusion could equally be drawn from our report that what is needed is an increase in budgetary aid rather than its withdrawal. Moreover, it should be noted that French budgetary support for fee exemption has been resumed and, moreover, after the national conference that put all of the problems on the table.

## "By focusing on the problems, you are playing into the hands of the opponents of fee exemption"

As researchers, our basic position was to distrust our ideological preferences (as citizens) and to try as much as possible to be neutral. We did not take sides and do not want to be associated with either camps. Above all, we want to report on a complex reality in the most rigorous and plausible manner possible.

That having been said, we cannot prevent anyone from using our findings. Given that the majority of the healthcare personnel oppose fee exemption (cf. Touré & Sanogo, in this issue), it is true that they can find arguments in our reports that support the cost-recovery model. However, the same applies to numerous supporters of universal health coverage who are upset with the fact that the fee exemption system is riddled with inconsistencies and believe that the fee exemption measures should be carefully prepared and solidly supported if the actual principle behind them is not to be discredited. They too can find arguments in our reports in support of better developed, better financed and better implemented fee exemption policies.

Let us take an example. In 2008, a 'pro-exemption' NGO, MDM (Médecins du Monde), extolled the virtues of the fee exemption policies without reservation and without attaching any importance to their detrimental side effects, writing that "the introduction of fee-exemption had a positive structural effect on the Nigerien health system" [6], and was still laudatory three years later [7]. But in 2012 MDM seemed to have taken (among other factors) our work into account (our findings have been notified previously to this NGO) [8] : "*However, we also want to emphasise that the introduction of these exemption policies should in no way be detrimental to the quality of care. In many countries, due to lack of preparation and/or any genuine political will, the decision to provide free access to healthcare has soon come up against huge difficulties in implementation with the only final result being the further destabilisation of health systems that are already largely failing. Initially made to improve access to healthcare, the hasty introduction of payment exemption has sometimes ended in the opposite situation by increasing drug shortages and/or de-motivation of health workers. Médecins du Monde wishes to reiterate that the move to 'free' healthcare is a structural reform which demands serious planning in advance of its adoption*". However, the 'camouflage culture' prevalent in both most NGOs and African administrations did not disappear either: a manager from the same NGO recently stated with respect to LASDEL's findings "*You shot down the fee exemption policy. I don't understand what is the intention here*!" We experienced similar attacks from some managers of Help, another NGO that, like MDM, campaigned for fee exemption and ran a pilot project in Niger.

## "You should focus on what works and not on what doesn't work"

Of course, the two perspectives are complementary and there is nothing to be gained from pitting them against each other. The instruction to focus solely on the positive aspects cannot be accepted. In social science a study should take both 'what works' and 'what doesn't work' into account.

On the one hand, identifying the aspects of a public policy that do not work is indispensable if there is any desire to improve it. The study of 'implementation gaps' is imperative. Adequate reform is impossible without the diagnosis of problems. Anthropology is supposed to provide a picture of the reference reality that is as complete as possible, sticking to its complexity as much as possible, without fear or favour [9]. Of course, this will upset some people: those who adopt the head-in-the-sand policy, those who wish to leave things as they are as they benefit from the status quo, and those who perceive all criticism of their service as a personal attack. However, it is difficult to deny the need for far-reaching reform demanded by the users and most clear-headed health personnel.

On the other hand, it is equally helpful to analyse the 'good practices' and document the 'success stories'. With qualitative approaches, this is a more difficult and complex task than it might seem. However, we consider it part of our mission as researchers and we do our best to accomplish it. Hence, with the support of the Belgian cooperation, LASDEL developed a research programme on 'reformers' in the health sector in Niger in the early 2000s, which, unfortunately, was terminated half way through the programme by the aid agency without any explanation.

## "The comments and behaviour you report are not representative. These are exceptions which you blow up out of all proportion"

Because of a public health culture in West Africa which is still highly epidemiological and not very open to the social sciences, many healthcare personnel object to our studies that they are 'non-scientific' as they 'do not contain any figures'. It is true that qualitative research is not based on statistical representativeness. This is its weakness. However, it is compensated for by numerous advantages [9] and it should be noted that graphs do not have a monopoly on either rigour or scientific quality and sometimes merely present the illusion thereof. Anyway, we are not afraid of numbers and we also make use of questionnaires (as was the case for our programme in Mali) [5], reviews, inventories and systematic document studies for our investigations.

Moreover, when using qualitative methods, we carry out endless cross-checks between interviews themselves, and between interviews and observations, repeated cross-referencing between our own data and the other available data, constant comparisons between different investigation sites, and repeated toing and froing between the production of data and their analysis. When we support our analysis using a quotation from an interview, it is representative of multiple comments that were made to us along the same lines. In other words, we report strong trends and current situations while paying great attention to diversity and variants. Our case studies are not rare exceptions; they reflect significant situations characterized by habitual processes. The practices we describe are central to and not at the margins of the everyday functioning of healthcare systems. For us, a study involves hundreds and sometimes thousands of in-depth interviews, dozens and sometimes hundreds of systematic observations. To the same extent as quantitative studies, but on another level, these provide 'convincing data' (evidence) for the famous 'evidence-based policies' which are so much on the agenda now.

Such mixed methods, in other words the combination of qualitative and quantitative measures as tested in this research programme, definitely represent the future of the social sciences and public health, especially for health policy and systems research (cf. Ridde & Olivier de Sardan, in this issue).

## "What you say is not new, we already knew about it"

In one sense, this is true. Our analyses are not 'scoops' or sudden revelations. The actors in the field and various experts close to the field are aware of the existence of the problems we describe to a greater or lesser extent.

However, for the most part these problems do not feature in the public arena and they are not rigorously documented. In other words, our research has the merit of producing robust, detailed, cross-checked and verifiable data on these problems that were not previously available. Hence, it makes it possible to put the problems 'on the table', to discuss them using solid data and not on the basis of rumours or impressions. It provides rigorous descriptions and serious case studies. It causes them challenges to be taken into consideration while a certain omertà had previously prevailed in relation to them.

In effect a double discourse prevails in countries "under the aid regime" [10] the official 'speak', on the one hand, and the private one, on the other.

The official speak, that of the meetings and encounters with cooperation agencies and international organizations, that of the public discourse, the innumerable workshops and seminars funded by the TFPs (Technical and Financial Partners), is agreed on, sterilized and far removed from reality. It is doublespeak (i.e. obfuscation) which oils the aid machinery and protects the routine of the local bureaucracies. People behave as if the figures presented in the reports were true, the activities had really been carried out, the quality of services were guaranteed, the official rules were respected, as though racketeering, informal privatization, absenteeism, and the deplorable mismanagement of human, material and financial resources did not exist.

Away from the TFPs, the private 'speak' is quite different. Everyone knows the everyday ruses, tricks and arrangements, and everyone jokes about them, even if this involves poking fun at the overzealous official who refuses to resort to them. In the course of informal encounters, it was not even unusual to hear the people who are most hostile to our studies describing with relish the details of this stark reality which they refuse to allow to be quoted in public. For instance a manager from the Ministry of Health in Niger told us during the preparation of the national conference on fee exemption: "Everything you say is true. People just don't want to hear you say it in public. We discussed this at our service in your absence. Everyone agrees that what you are doing is good" (comment recorded by Mahaman Moha). This is reminiscent of a comment made by a Nigerien doctor during another study by LASDEL: "The LASDEL report says out loud what we whisper to each other and do not dare to say" [11].

The two 'speaks' do not communicate with each other. No health worker, or as good as no one, would allude publicly to the multiple illicit practices that prevail in their service. No NGO worker, or as good as no one, would draw their employer's attention to the abuses, implementation gaps or mismanagements suffered by the project which they have the task of promoting. This is not even necessarily the effect of a deliberate desire to hide things. This type of reminder of the reality based on the 'private language' is simply not considered relevant or appropriate in the arena of the public language. This would be breaking a tacit rule of the game, that can be even be observed sometimes in the press: while Malian journalists ignore the difficulties associated with the implementation of fee exemption at the point of service delivery, their editors refer to it in private discussions [12].

Using its qualitative techniques, LASDEL *collects the private speak and transmits it to the public sphere through its analyses *(cf. Ridde & Olivier de Sardan, in this issue). Moreover, it does this in a way that is as rigorous, meticulously documented, non-partisan and impartial as possible. It is never our intention to denigrate a given policy, and even less to launch personal attacks. What we do intend is to provide a serious account of a reality about which nobody speaks officially and to create the conditions for an expert debate and a public debate. While the actors on the ground are all more or less familiar with this reality, we confirmed on various occasions, to our considerable surprise, that the national political leaders and high-level technocrats appear to ignore it, at least in part. It is true that they never go to a local health centre or even a public hospital for treatment. They only show up at health facilities for openings or meticulously staged official visits.

## Conclusion

Admittedly, in the case of Niger where the problems were most acute, we had the feeling that our findings were not in the least welcome from the perspective of the Ministry of Health and other public authorities - at least during the first three years of our research. We communicated them through a widely distributed policy brief. Also the 'pro-fee-exemption' NGOs were embarrassed for a long time by the demonstration of the negative side effects of a policy they had ardently promoted.

But the tide gradually turned. In particular the national conference on fee exemption held in Niamey in March 2012 was a crucial milestone. Following its preparation in the form of a briefing by two members of our team acting as consultants (Valéry Ridde assisted by Mahaman Moha, a researcher at LASDEL), we played an important role at the conference by presenting four policy briefs, two public lectures and various contributions to panels. For the first time, people publicly spoke about the numerous problems associated with the fee exemption policy, and the healthcare personnel present largely confirmed and even supplemented our findings [13]. This shows that the effects of the research we carry out should be assessed in the medium to long term. This is another difference with the work carried out by consultants, which is usually commissioned and carried out as a matter of urgency and is very often limited in its level of detail and life-span as a result [14].

However, it is possible to draw two other conclusions which appear to be particularly important.

First, the field of health remains an area that involves strong economic, political and ideological challenges, in which personnel are not free to speak and remain constrained as much by a strong sense of hierarchy as by various 'camouflage' strategies, and in which the views of users are not collected let alone taken into account. Doublespeak prevails in official institutions (ministries, international organizations, aid agencies, national NGOs and international NGOs). Although international NGOs sometimes directly oppose national policies - cf. Médecins Sans Frontières in Niger during the food crisis of 2005 - they also have their own doublespeak and are very reticent when research highlights their inadequacies [15]. For its part, the press generally acts as the spokesperson of these official institutions [12]. However, it is above all the reports and publications produced by researchers, if they broach the topic of the implementation gap, that can act as levers to open up the debates on the every realities experienced by health service personnel and users rather than the bureaucratic fictions, dubious statistics, self-justificatory declarations or formal instructions as is most often the case at present [14]. It is difficult to see how the healthcare system can be improved and better quality of service provided to the users without starting from a rigorous diagnosis of these usually concealed realities.

2. Second, the healthcare personnel were seen to react to our findings in two very different ways. Some of them were sceptical [16], denied them or tried to discredit them (often by referring to a caricature-like view of scientific quality that was reduced to statistics) because they believe that these realities do not have any place in the public discourse and/or because they do not want to change the current situation which often benefits them. These are, we dare say, the 'conservatives'. Others, in contrast, were delighted to finally see these problems being aired in the open and wanted to support our analyses to make change happen. We can label the latter as 'reformers' who want to promote the public interest.

This is where we leave behind our quest for neutrality as researchers and don our citizens' hats [9]. In the course of its implementation, our research is as impartial and 'objective' as possible. But we select topics that involve real political and social issues at the outset and we hope that our findings will be used by reformers in their - mostly latent but sometimes open - battle with the conservative force.

## List of abbreviations

ACT : Artemisinin-based combination therapy

AFD : Agence Française de Développement

ASACO : Association de Santé Communautaire

LASDEL : Laboratoire d'études et de recherches sur les dynamiques sociales et le développement local

MDM : Médecins du Monde

NGO : Non Governmental Organisation

TFP: Technical and Financial Partner

WHO: World Health Organization

## Competing interests

None

## Authors' contributions

JPOS conceived the idea, wrote the draft and final version of the manuscript.

## Declarations

This article has been modified from the chapter **Les exemptions de paiement: controverses et malentendus autour d'une recherche. Les chercheurs et le débat citoyen** by J.-P. Olivier de Sardan [17] in the book *Une politique publique de santé et ses contradictions. La gratuité des soins au Burkina Faso, au Mali et au Niger*, J.-P. Olivier de Sardan and V. Ridde (eds), 2014, Karthala, Paris, with the permission of the publisher. The publication of this supplement was funded by a grant from International Development Research Centre (IDRC), Ottawa, Canada.

This article has been published as part of *BMC Health Services *Research Volume 15 Supplement 3, 2015: User Fee Exemption Policies. The full contents of the supplement are available online at http://www.biomedcentral.com/bmchealthservres/supplements/15/S3.
